# Effect of different shapes of steel fibers and palygorskite-nanofibers on performance of ultra-high-performance concrete

**DOI:** 10.1038/s41598-024-59020-8

**Published:** 2024-04-08

**Authors:** Yingying Huang, Dewen Kong, Yi Li, Shenghui Zhou, Jing Shu, Bing wu

**Affiliations:** Guizhou University, Guiyang, China

**Keywords:** Ultra-high-performance concrete (UHPC), Palygorskite, Straight-steel fiber, Hooked-end-steel fiber, Mechanical properties, Nanoscale materials, Materials science, Mechanical properties

## Abstract

Herein, a practical ultra-high-performance concrete (UHPC) was created by adding two different shapes of steel fibers and curing them at ambient temperature using palygorskite-nanofiber (PN) as the modifier. The compressive strength, flexural strength, water absorption capacity, and porosity were analyzed to determine the effects of the steel fibers and PNs on the UHPC mechanical and physical properties. The steel fibers and PNs were found to improve these properties. The UHPC mechanical properties were outstanding at 1.5% fiber dosage, while physical properties were excellent at 1.0% fiber dosage. The mechanical and physical characteristics of UHPC were preferably at a PN dosage of 0.2% and the fiber dosage of 1.0%. The compressive and flexural strengths of straight-steel-fiber UHPC were 145.57 and 19.67 MPa, respectively, i.e., 42.0 and 109.4% higher than those of the reference specimens (i.e., those without fibers or PNs); the water absorption capacity and porosity decreased by 50.1 and 60.7%, respectively. The compressive and flexural strengths of hooked-end-steel-fiber UHPC were 18.3 and 96.0% higher than those of the reference specimens, respectively, and the water absorption capacity and porosity decreased by 43.2 and 29.8%, respectively. These results could provide vital information for the promotion and practical application of UHPC.

## Introduction

Concrete is widely used in construction projects because of its low cost and the easy availability of raw materials^1,2^. However, the drawbacks of self-weight and poor toughness of ordinary concrete impact its life and usage, and also limit its application in high-performance projects^3–5^. Therefore, ultra-high-performance concrete (UHPC) has been developed as an innovative cement-based material with ultra-high durability and superior mechanical properties^6–8^.

The preparation of UHPC usually involves an extremely low water–binder ratio, very fine aggregates, a high cement content, and the removal of coarse aggregates^9–11^. Therefore, the cost of UHPC depends on the selected raw materials, and the risk of early shrinkage-induced cracking caused by the rejection of coarse aggregates cannot be ignored. Currently, the performance optimization of UHPC materials is mainly carried out from the following aspects^12–14^. First, supplementary cementitious materials (SCMs) are often used to replace a part of the cement, thus reducing the cement content of UHPC to achieve green and low-cost UHPC production. Huang et al^15^. explored the effects of common dopants such as limestone powder, fly ash, and granulated blast furnace slag on the macroscopic properties and microstructure of UHPC, and reported that the above materials at appropriate dosages improve the workability and mechanical properties of UHPC because these materials effectively fill the pore structure of UHPC at the microscopic level. Then, Zhuang et al^16^. successfully prepared green UHPC with a compressive strength of > 120 MPa by using copper slag, which is an industrial waste. Then, Park^17^ reviewed the effects of SCMs such as slag, fly ash, limestone, and metakaolin on the macroscopic properties of UHPC in terms of both material properties and environmental impacts, noting that all these SCMs aid in satisfying the UHPC-compressive-strength requirements.

The use of appropriate raw materials or the addition of expansion agents can produce UHPC with a wide range of applications^18,19^. However, the properties of the UHPC prepared by replacing steel fibers with other organic fiber blends were poor, which indicates that steel fibers are an indispensable raw material^20^. Previous studies have indicated that optimizing the raw materials and curing methods of conventional UHPC based on achieving the tightest packing of particles possible improves both the cost-effectiveness and performance of UHPC. For example, Faried et al^21^. and Mostafa et al^22^. reported that standard conservation (SC) is the best curing method that is economical and substantially improves UHPC strength. Other researchers^23–25^ have found that the preparation of UHPC using mechanized sand (MS), which is obtained by the mechanical destruction and screening of large blocks of rock, instead of natural sand (e.g., silica sand^26^, river sand^27^, and quartz sand^28^) through a mixed design is more environmentally friendly^29^. Furthermore, the MS UHPC produced at room temperature exhibits the same mechanical properties and frost resistance as natural-sand UHPC^30^. Nevertheless, the mechanical strength of MS is inherently low, so the production of MS UHPC with both workability and required mechanical properties is difficult. In contrast, the small coarse silicate-type basalt aggregates are commonly used in the preparation of high-strength concrete due to their excellent mechanical properties^31,32^. In summary, the usage of MS and basalt instead of natural sand for UHPC preparation enables the balancing of the cost with the realized performance.

Apart from these optimization approaches, nanomaterials have also emerged as promising additives for improving the compactness of the UHPC matrix and for improving various other properties^33^. In previous studies^34–36^, the effectiveness of nanomaterials has been confirmed. These materials enhance the compactness of the microstructure of cementitious materials, thus improving the interfacial transition zone between cement paste and aggregate in the UHPC system and subsequently improving the mechanical properties of UHPC. However, commonly utilized nanomaterials such as SiO_2_ and CaCO_3_ nanoparticles and carbon nanotubes are very expensive. Hence, a suitable cost-effective nanomaterial is urgently needed. Palygorskite—an inexpensive silicate mineral material—is capable of meeting these requirements. It is commonly used to improve cement-based materials because of its natural one-dimensional nanorod structure and large surface area^37,38^. Kawashima et al^39^. simulated the casting process of cement slurry by incorporating high-purity palygorskite-nanofiber (PN) and discovered that the shear damage recovery rate of concrete with 0.5 wt% palygorskite was 60% higher than that before the addition. Qu et al^40^. added modified purified palygorskite to stabilized cement aggregates; they found that the addition of palygorskite significantly improves cement compressive strength and the effect of palygorskite addition was better than that of montmorillonite and kaolinite addition. Zhan et al^41^. observed that the addition of palygorskite enhanced the toughness of PVA-fiber-cement-based materials. The results of Yan et al^42^. showed that PN could significantly improve the mechanical properties of recycled concrete. Thus, PN can significantly enhance the properties of cementitious materials. Besides the above studies, palygorskite, as a new silicate material, has been used to prepare mineral wool acoustic panels or to enhance the active filler. However, only a few studies have focused on the performance of UHPC modified with PN.

Therefore, it is necessary to develop studies on the effect of PN on the properties of UHPC. Based on above, in this study, UHPC is prepared by using PN as the modifier, by replacing natural sand with MS and basalt. Furthermore, curing at ambient temperature is performed, and the effects of different types of steel fibers and PN addition on the performance of UHPC are analyzed. The aim of this study is to prepare a UHPC material with good practicality and provide the technical knowledge necessary for the popularization of UHPC.

The total amount of cementitious material used for the UHPC prepared in this study was only 635 kg/m^3^, and 145.57 MPa of UHPC was obtained with the incorporation of 1% by volume of straight steel fibers as well as 0.2% by mass of PN, which was found to be more economical and superior as compared to the scholars such as Li^43^, Ding^44^, and Fan^45^. In addition, the materials used in this paper are all less than 5 mm in diameter, so they can be used for thin members that resist bending without traditional steel reinforcement, and their great waterproofing properties can also be used for the decoration of the exterior walls of houses. A utility patent has been filed for this research, and subsequent research will be evaluated for practical applications.

## Experimental program

### Raw materials

**Figure 1:**
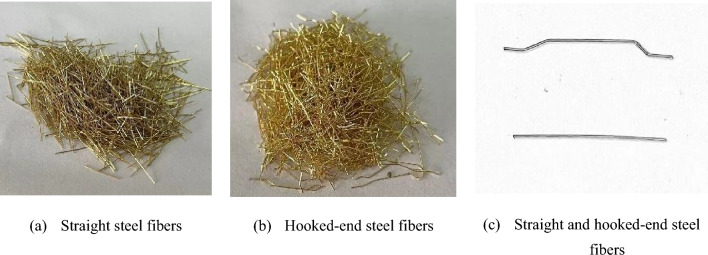
Straight and Hooked-end steel fibers.

**Figure 2:**
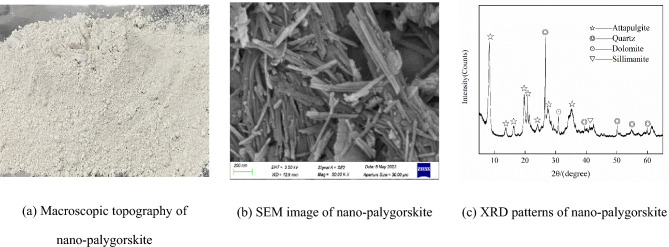
Macroscopic topography, SEM image, and XRD patterns of nano-palygorskite.

1. P·II 52.5 R cement produced by Guiyang Conuo Cement Factory was used, and its main properties are shown in Table 1.
2. Silica fume was obtained from Gongyi Baichuan Water Treatment Co. Its capacitance is 16 × 104 cm^2^/g, and its main properties are listed in Table 2.
3. Basalt crushed stone sample with 3–5 mm particle size and an apparent density of 2680 kg/m^3^ was procured from Qingzhen Jurong Building Material Development Co.
4. MS was obtained from Guizhou Lianjiangyuan Building Materials Co., and it had a particle size of 0–4.75 mm, mud content of 0.9%, and fineness modulus of 2.6.
5. A polycarboxylate-based water-reducing agent with a water reduction rate of 35% and a solid content of 40% was provided by Hunan Zhongyan Co.
6. Straight and hooked-end steel fibers were used. Their macroscopic shape is displayed in Fig. 1, and the main characteristics are displayed in Table 3. As seen in Fig. 1, the two types of steel fibers with different shapes are gold-plated; further, the middle part of the hooked-end steel fibers is straight, and the two ends are hooked-end.
7. The PN was obtained from Changzhou Dingbang Mineral Products Co. The macroscopic morphology, SEM image, and XRD and XRD patterns are shown in Fig. 2. Figure 2 shows that PN exists as a white powder, and the powder particles are one-dimensional crystalline nanorods. The XRD patterns show a high SiO_2_ content in the PNs, which facilitates the production of more hydration products from silicate cement. The chemical composition and performance index of PN are shown in Table 4.
8. The water used is the laboratory tap water.

**Table 1:** Properties of cement.

Cement mark	Initial setting time/min	Final setting time/min	Compressive strength/MPa		Flexural strength/MPa	
			3d	28d	3d	28d
P·II52.5 R	188	236	39.8	60.6	6.9	9.1

**Table 2:** Main properties of silica fume.

	SiO_2_/%	Total alkali content /%	Firing loss/%	Water demand ratio/%	Water content/%	Activity index/%
Silica fume	92.2	1.3	3.0	107	1.8	116

**Table 3:** Technical specifications of steel fibers.

Steel fiber	Tensile strength /MPa	Diameter/mm	Length/mm	Aspect ratio
Straight	2800	0.20	13	65
Hooked-end	2800	0.20	15	75

**Table 4:** Chemical composition of nano-palygorskite (wt%).

Material	SiO_2_	Al_2_O_3_	CaO	Fe_2_O_3_	MgO	K_2_O	SO_3_	Na_2_O	Lol
PN	62.9	12.0	3.01	7.02	11.8	1.52	0.11	0.16	1.48

### Experimental design

According to the research of numerous scholars, nanomaterials are found to affect the flowability, mechanical properties, physical properties and micro-mechanisms of UHPC. However, based on the results of the previous experiments we carried out, revealed that the effect on the flowability of UHPC is not significant because of the small amount of PN added to UHPC. Flowability of UHPC with the maximum amount of PN added was only reduced by 10 mm compared to the unadded one. Therefore, for this reason and due to the length of the article, this paper focuses on describing the physical and mechanical properties of UHPC.

Based on the complete method for calculating the composition and properties of cementitious materials^46^, the mix proportion for this study was determined, as shown in Table 5, to explore the effects of different steel fiber shapes, different steel fiber dosages, and PN on the mechanical and physical properties of UHPC. Among them, the steel fiber and PN contents are presented as the volume percentage and mass percentage of the total cementitious material, respectively.

**Table 5:** Mix proportion of prepared UHPC (kg/m^3^).

Notation	Cement	Silica fume	Machine sand	Basalt	Water–binder Ratio	Water reducing agent	Steel fibers (%)	PN (%)
UJ	552	83	659	754	0.17	3%	0	0
Us0.5							0.5	
Us1							1	
Us1.5							1.5	
Us1p0.1							1	0.1
Us1p0.2								0.2
Us1p0.3								0.3
Uh0.5							0.5	0
Uh1							1	
Uh1.5							1.5	
Uh1p0.1							1	0.1
Uh1p0.2								0.2
Uh1p0.3								0.3

*UJ: reference specimen; *Us1p0.2: control specimen containing 1% straight fiber and 0.2% PN; *Uh1p0.2: control specimen containing 1% hooked-end fibers and 0.2% PNs. Each group involves three specimens per group for investigating compressive, flexural, and physical properties. There are a total of 13 groups and 117 specimens.

### Specimen preparation

**Figure 3:**
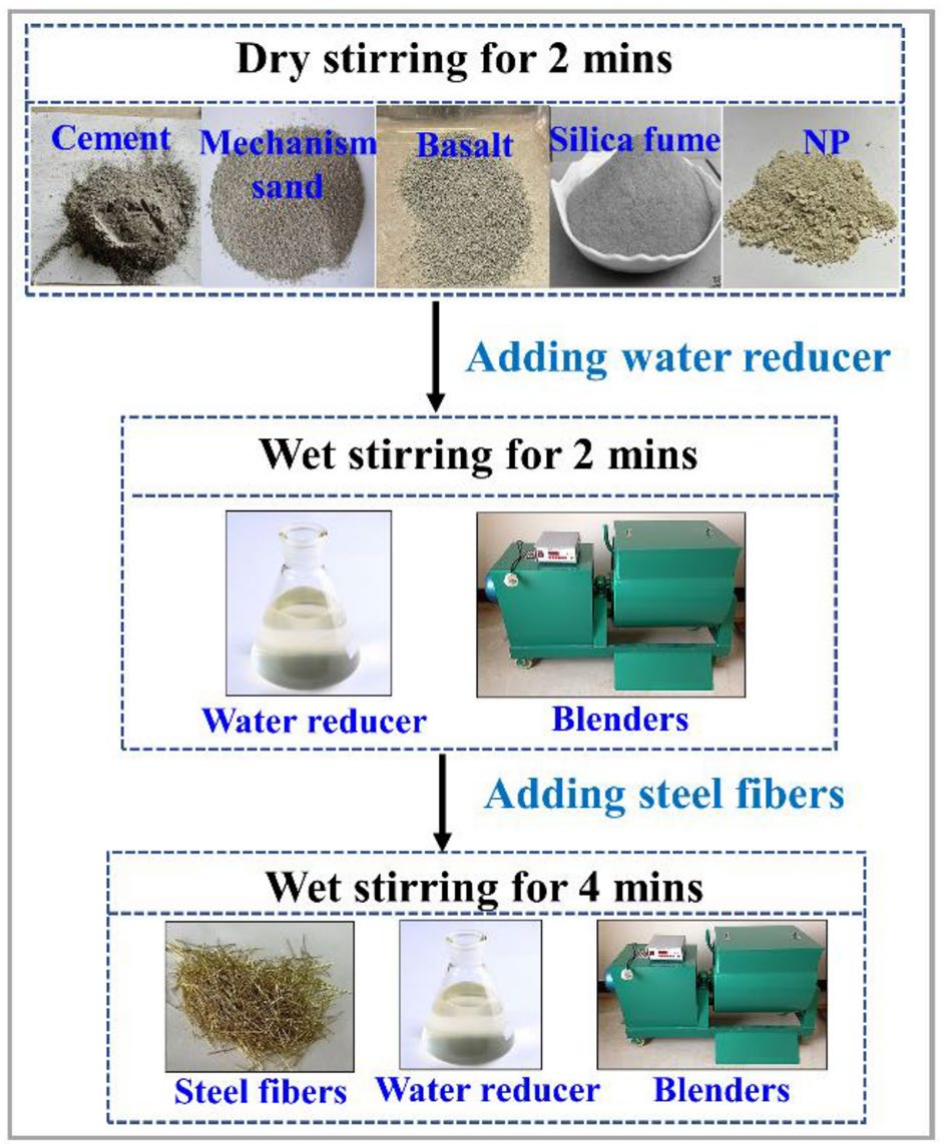
Mixing process of ultra-high-performance concrete.

The cement, silica fume, MS, basalt, and PN were mixed dry for 120 s after weighing. Then, water and 70% of the water-reducing agent were added and mixed for 60 s. Thereafter, the remaining water-reducing agent was added and mixed for 60 s. After the matrix was wet and adhesive, steel fibers were evenly spread and mixed in for 240 s. Finally, the freshly mixed slurry was loaded into a steel mold and vibrated for 60 s to complete the molding. The mixing process of UHPC is shown in Fig. 3. The fresh pastes were cast into 100 × 100 × 100 mm and 100 × 100 × 400 mm cubic steel molds for compressive strength and flexural strength tests, respectively. To test the water absorption capacity and porosity, the fresh pastes were cast into a 100 × 100 × 100 mm plastic mold. The molded specimens were demolded approximately 24 h after casting and then cured at room temperature of (20 ± 2 °C). The compressive and flexural strength were tested after 7, 14, and 28 days, and the water absorption capacity and porosity were tested after 28 days.

### Testing methods

This study referred to Li's study^47^ and tested the compressive and flexural strength of UHPC based on ASTM C109/C109M-02 (standard test method for compressive strength of hydraulic cement mortars) and ASTM C78/C78M-10e1 (standard test method for flexural strength of concrete), respectively. WHY-2000 universal testing machine was used for testing, and the compressive strength and flexural strength hair load rates were 1.2 MPa/s and 0.5 MPa/s, respectively. Each of the 13 groups listed in Table 5 had three samples, and the average value of the valid data from each group was selected as the final test result.

We refer to Zhus article^48^ and the international standard ASTM C1585-2013 to test the water absorption and porosity of UHPC. The water absorption capacity and porosity were tested as follows: (a) The specimens were cured for 28 days, then placed into a blast drying oven at a temperature of (105 ± 5 °C) and dried for 48 h. The drying time of the specimen should not be less than 48 h until the two consecutive 24 h intervals of the quality change is less than the smaller value of 0.2%; stop drying, and record the last specimen quality, which should be accurate to 0.1 g, expressed in M_d_. (b) The specimens were cured for 28 days, then soaked in water for 24 h, removed, wiped with a damp towel, and weighed. Next, they were soaked for another 24 h, taken out, wiped with a wrung-out wet towel, and weighed. The specimen soaking time should not be less than 48 h until the two consecutive 24 h interval of quality change is less than 0.2% of the larger value; stop soaking, and record the last specimen quality, which should be accurate to 0.1 g, expressed in M_s_. (c) The specimen after tested submerged in water, using the electronic hydrostatic balance to test its suspended mass, which should be accurate to 0.1 g, expressed in M_w_. Water absorption is obtained by subtracting the dry mass from the water saturated mass and dividing by the dry mass. Porosity is calculated by subtracting the dry mass from the saturated mass and dividing by the saturated mass minus the floating mass.

## Results and discussion

### Effect of steel fibers on physical properties of UHPC

**Figure 4:**
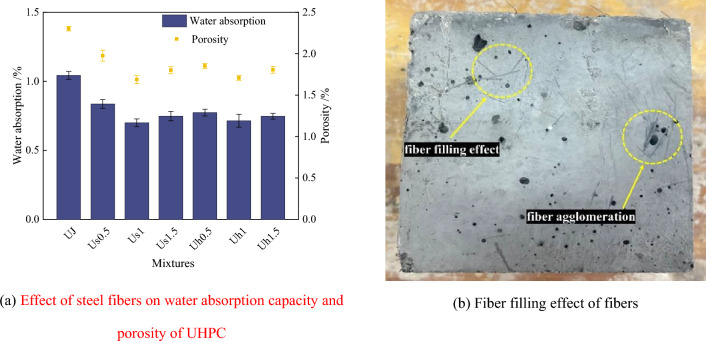
Effect of steel fiber on water absorption capacity and porosity of UHPC and fiber-filling effect.

Figure 4 displays the effects of straight and hooked-end steel fibers on the water absorption capacity and porosity of UHPC. As shown in Fig. 4a, the incorporation of steel fibers can significantly reduce the water absorption capacity and porosity of the UJ specimens. With the increase in the fiber dosage, the water absorption capacity and porosity of UHPC specimens with two different shapes of steel fibers decreased first and then increased. UHPC specimens with the two fibers exhibited the lowest water absorption capacity and porosity when the steel fiber dosage was 1%. The water absorption capacity and porosity of the Us1 group are the most negligible, measuring 0.699% and 1.688%, respectively. These values are 33.0% and 26.7% lower than those of the UJ group, respectively. The water absorption capacity and porosity of the Uh1 group are 0.714% and 1.707%, respectively, representing values 31.5% and 25.9% lower than those of the UJ group. This is due to the filling effects of straight and hooked fibers, which act as a skeleton in the matrix to help carry the load, resulting in a dense fiber network structure. This hindered the invasion of external moisture and the formation of cracks inside the matrix. Thus, fibers can effectively reduce the water absorption capacity and porosity of UHPC. The effect of fiber filling is exhibited in Fig. 4b.

However, notably, when the fiber dosage is 0.5 and 1.5%, the water absorption capacity and porosity of the hooked-end fiber UHPC are less than those of the straight-fiber UHPC. This is attributed to the end hooks of the hooked-end fibers resulting in a stronger mechanical bite into the substrate and thereby inhibiting the intrusion of external moisture, thus reducing the surface porosity of the UHPC. When the fiber dosage is increased, the fibers rotate and flow in the direction perpendicular to the cement slurry at the edge of the matrix, which causes a decrease in the matrix flow rate^49^, resulting in an increase in the fiber lap and winding rate. Therefore, the number of fibers that fill the surface pores of the specimen is limited. Thus, a further increase in the number of hooked-end fibers becomes no longer beneficial to reduce the water absorption capacity and porosity of UHPC.

### Effect of steel fibers on mechanical properties of UHPC

**Figure 5:**
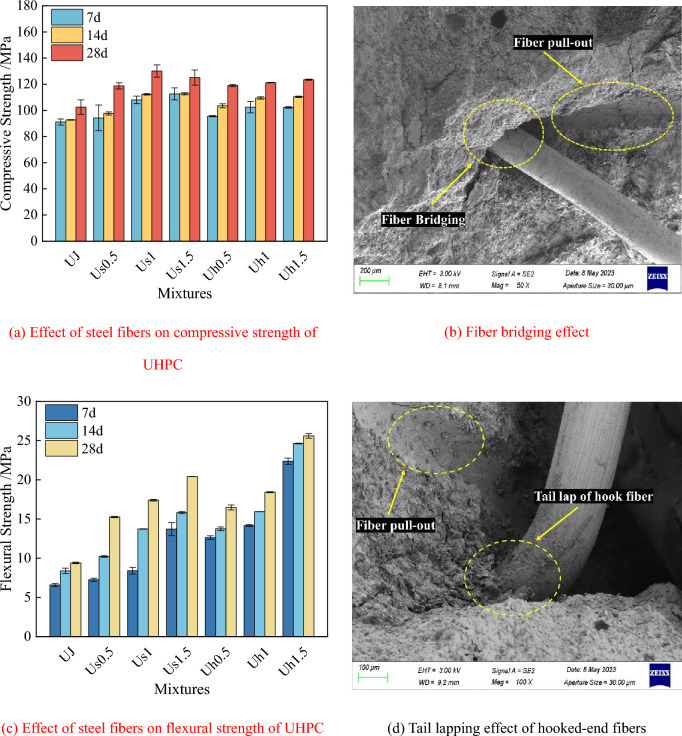
Effect of steel fibers on mechanical property of UHPC.

Figure 5 shows the combined influence of straight and hooked-end steel fibers on the compressive strength and flexural strength of UHPC through the “bridging effect” (i.e., steel fibers connecting different parts of the matrix together like a bridge) of the two types of fibers, as visualized by microscopy. According to Fig. 5, the incorporation of the steel fibers effectively improves the compressive and flexural strength of UHPC. Figure 5a shows that the 7 and 14 d compressive strength of straight-steel fiber UHPC increases continuously with the fiber dosage, while the 28 d compressive strength first increases and then decreases. The 7, 14, and 28 d compressive strength of hooked-end steel fiber UHPC gradually increases with the fiber dosage. This is owing to the appropriate steel fibers undergoing a valid penetration of the “fiber bridge” inside the UHPC, and the overlapping effect of the two types of fibers inside the matrix is presented in Fig. 5b, d. The addition of steel fibers can effectively reduce the porosity in the matrix. However, the addition of a 1.5% steel fiber content will increase the probability of fiber agglomeration, and the overlap between fibers will prevent the discharge of bubbles. Ultimately, the reduction in the internal stacking density limits the improvement of UHPC compressive strength. Therefore, when the steel fiber dosage is 1%, the Us1 group has the superior 28 d compressive strength (130.13 MPa), 27.0% higher than that of the UJ group (102.5 MPa). Notably, the hooked-end steel fibers consistently increase the compressive strength of UHPC under the same volumetric dosage. This is because, for the same fiber content, the number of straight fibers with a large length-to-diameter ratio exceeds that of hooked-end fibers^50^. Thus, the 1.5% content of straight fibers is not conducive to the improvement of UHPC compressive strength. On the contrary, the number of hooked-end fibers is insufficient at a 1.5% dosage, resulting in a slight improvement in the compressive strength of the UHPC. However, overall, the compressive strength of hooked-end fiber UHPC is lower than that of straight-fiber UHPC. At the same time, the ends of hooked-end fibers tend to become entangled, so these fibers are not as uniformly dispersed inside the matrix as straight fibers. Consequently, the increase in the compressive strength of UHPC is lower than that in the case of straight fibers.

In addition, Fig. 5c shows that both straight and hooked-end steel fibers can improve the flexural strength of UHPC, which increases with the fiber dosage, but the hooked-end steel fibers are more effective in increasing flexural strength. The ends of the hooked-end fibers result in high mechanical bite force and pull-out force^51,52^, thus increasing the UHPC’s ability to withstand external loads. In addition, according to the theory of fiber spacing, which states that the initial crack strength of fiber-reinforced concrete is inversely proportional to the square root of fiber spacing^53^, the presence of the end hooks reduces the distance between fibers, hindering the expansion of cracks. Furthermore, fiber pull-out failure usually occurs during the flexural test. However, in the case of hooked-end fibers, the interconnected network structure is less likely to experience fiber pull out than the fiber network composed of straight steel fiber; this also contributes to a considerable extent to the enhanced flexural strength of UHPC with the addition of the hooked-end steel fibers. The most supplementary cementitious materials (SCMs) mechanical properties of straight-steel-fiber UHPC and hooked-end-steel fiber UHPC are obtained with a fiber dosage of 1.5%. The compressive strength and flexural strength of straight-steel-fiber UHPC are 125.13 and 20.41 MPa, respectively, and those of hooked-end steel fiber UHPC are 123.50 and 25.60 MPa, respectively; these four values are 22.1, 117.3, 20.5, and 172.6% higher than those of the UJ group. In general, the mechanical properties of UHPC are better at 1.5% fiber dosage, while the physical properties are preferably when the fiber dosage is 1.0%. Considering the comprehensive performance and economic aspects, 1% fiber dosage is selected as the most superior. Thus, the subsequent tests are performed with the samples with a 1% steel fiber dosage.

### Effect of PN on physical properties of UHPC

**Figure 6:**
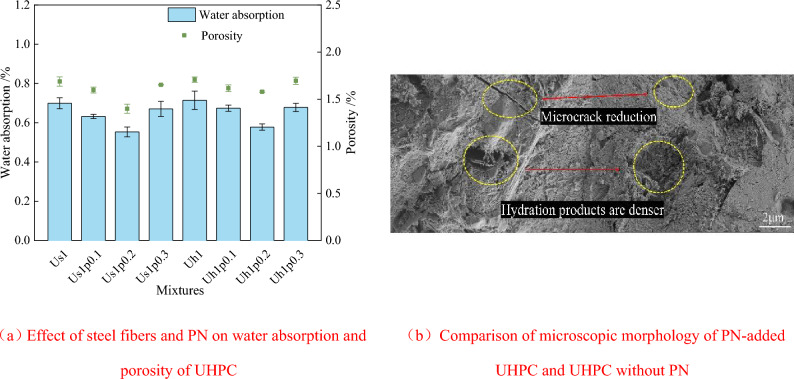
Effect of steel fibers and PN on physical property of UHPC and comparison of microscopic morphology.

Figure 6 reveals the influence of PN dosage on the water absorption capacity and porosity of UHPC. From subsection 3.1, we know that UHPC physical properties are excellent with a 1% steel fiber dosage. Hence, specimens with 1% fiber dosage are used for this part of the study. As shown in Fig. 6a, the addition of PN reduces the water absorption capacity and porosity of the control group (Us1 and Uh1) to a certain extent. When the PN dose is increased, the changing trend of water absorption capacity and porosity of UHPC is similar to that observed with the increase in steel fiber content. Specifically, when 0.2% PN is added, the water absorption capacity and porosity of the Us1p0.2 specimens are lowest—0.553 and 1.399%, which are 0.146 and 0.289% less than those of the Us1 specimens (0.699 and 1.688%). Next, the water absorption capacity and porosity of the Uh1p0.2 specimens decline rapidly, and are 0.136 and 0.128% less than those of the Uh1 specimens (0.714 and 1.707%). In addition, from the comparison of the microscopic morphology of UHPC in Fig. 6b, it shows that the hydration products of UHPC with PN are more abundant and denser than the reference group, and have fewer microscale cracks, thus the densification of the hardened body is improved. This result indicated that the appropriate amount of PN promotes the generation of UHPC hydration products^42^, which, in turn, fills the micropores of the UHPC paste network.

The effect of PN addition on the water absorption capacity and porosity of UHPC is not evident when the PN dosage is increased further to 0.3%, and the water absorption capacity and porosity of Us1p0.3 and Uh1p0.3 specimens are the same as those of the control group. PN has a nano-channel structure that can store free water^54^. A higher PN content results in the absorption of a larger amount of free water inside the matrix and limit the diffusion of Ca^2+^ and OH^−^, thus delaying the hydration reaction inside UHPC. The ensuing problem of nanomaterial agglomeration will also restrict PN dispersion ability^55^, thereby limiting its contribution to the microstructure of the hardened body. This may introduce new structural defects, eventually leading to an insignificant degree of decrease in the water absorption capacity and porosity of UHPC at 0.3% PN dosage. In addition, the histogram in the Fig. 6 reveals that the water absorption capacity and porosity of the hooked-end fiber UHPC are generally higher than those of the straight-fiber UHPC. This can be ascribed to the greater water absorption capacity and the greater propensity for agglomeration of PN and also to the possibility of entangling the tail ends of hooked-end fibers. Thus, these inherent limitations of both PN and hooked-end fibers might diminish the effects of both materials.

### Effect of PN on mechanical properties of UHPC

**Figure 7:**
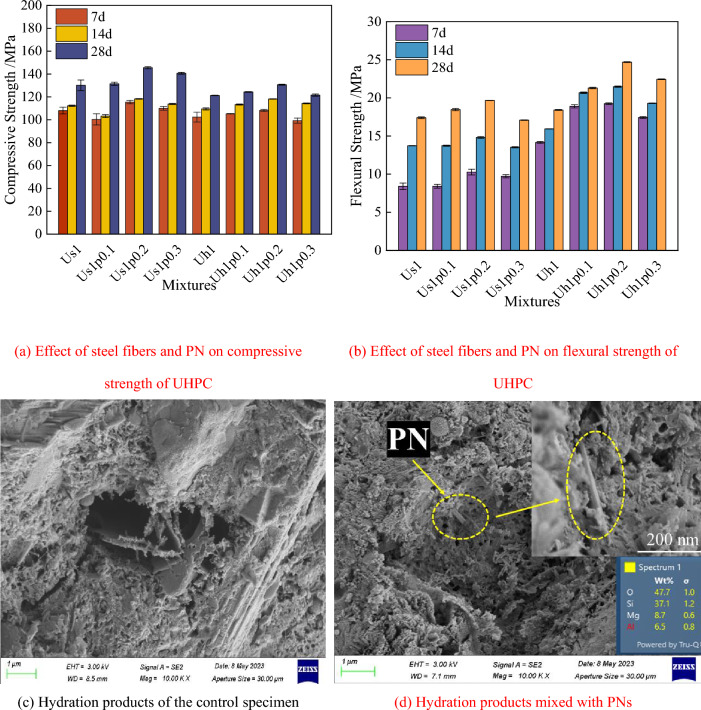
Effect of steel fibers and PNs on mechanical property of UHPC.

Figure 7 shows the effect of PN on the mechanical properties of UHPC and the SEM images of the hydration products of PNRUHPC. As observed in Fig. 7a, the compressive strength of straight-fiber UHPC with 0.1% PN at 7 and 14 d is slightly lower than that of the reference group, but at 28 d, it is slightly higher. Owing to the strong water absorption capacity of PN, despite the small PN amount, it absorbs part of the free water inside the matrix, resulting in insufficient hydration of the matrix within a short period and thus a slight decrease in the early strength of UHPC. As the PN dose is increased, the compressive strength of both straight-steel-fiber UHPC and hooked-end steel fiber UHPC first increases and then decreases. Following the addition of 0.2% PN, the 28 d compressive strength of the two UHPC specimens with different shapes of steel fibers shows a considerable enhancement: the compressive strength of the straight-steel-fiber UHPC and hooked-end steel fiber UHPC was 145.57 and 130.70 MPa, respectively, which was 15.44 and 9.43 MPa greater than those of the control group, respectively. Notably, Fig. 7b shows that the 0.2% PN dosage has the most prominent effect on the improvement of the flexural strength of UHPC. In addition, the flexural strength of straight-steel-fiber UHPC and hooked-end steel fiber UHPC is 19.67 and 24.69 MPa, respectively, which is 13.1 and 41.8% higher than those of the reference group, respectively. When PN is added, the change law of flexural strength of UHPC is similar to that of compressive strength.

The height of the comparative compressive and flexural strength histograms of the two steel fibers is shown in Fig. 7a, b. Interestingly, it was observed that the combined effect of PN and straight steel fiber could dramatically increase the 28 d compressive strength of UHPC; at the same time, the synergistic effect of PN and hooked-end steel fiber is more favorable to the 28 d flexural strength of UHPC. This is primarily due to the micro-filling effect (i.e., the incorporated PN fills the nanoscale pores inside the matrix) and the volcanic ash effect (i.e., the silica fumes react with Ca(OH)_2_ in a secondary hydration reaction to form a cementitious product that fills the cement stone structure) of PN promoting the generation of hydrated calcium silicate, thereby increasing the density of the interior of UHPC. Moreover, PN and steel fibers are fillers at the nanometer-scale and millimeter-scale. The combined effect of both improves the dense internal structure of UHPC and enhances the continuity between the hardened bodies, thereby amplifying the effect of steel fibers on the compressive and flexural properties of UHPC. The chemical formula of PN is (Mg, Al)5[(OH)2(Si, Al)8O20]· 8H2O, and a semi-quantitative analysis based on the EDS energy spectrum of Fig. 7d, identifying the material in the yellow circle in the figure is PN. A comparison of Fig. 7c, d also illustrate that the addition of PN increases the amount of hydration products of UHPC and the compactness of the matrix interior. In the case of low PN dosage, the micro lap effect of PN is not significant, and this reduces the early strength of UHPC. When the dosage is too high, the cohesion of the matrix increases, and local stress concentration readily occur, which will lead to the agglomeration of PN. Furthermore, because of the high-water absorption capacity of PN, the uptake of a large amount of free water inside the UHPC matrix occurs. This causes insufficient hydration reaction inside the UHPC and reduces the density of the hardened body, thereby limiting the improvement in mechanical properties. Hence, the optimum dosage of PN is 0.2% of the total mass of the cementitious material.

### Macroscopic failure mode of UHPC

**Figure 8:**
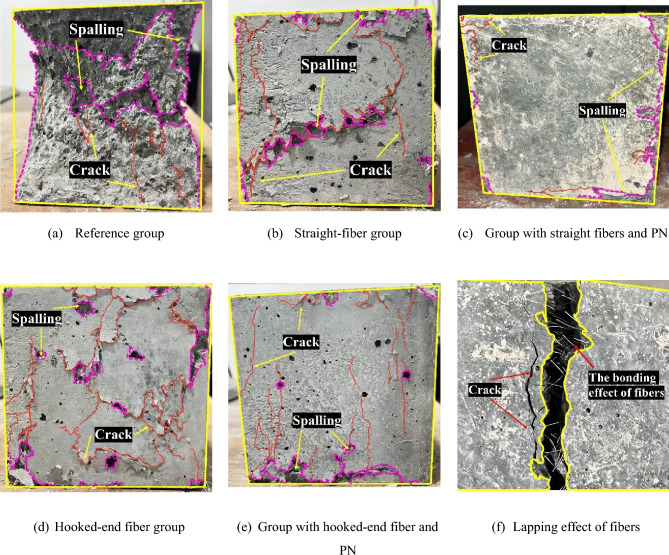
Macroscopic failure mode and fiber bonding of UHPC.

Figure 8 depicts the failure mode of the different concrete specimens and the effect of steel fiber lap. A comparison of Fig. 8a, b, c show that the reference group exhibits brittle damage with a large number of chips breaking off from the surface, and the specimen is deformed after the final crushing damage. With the addition of steel fibers, the damage of UHPC changes from brittle to ductile. The specimens do not undergo severe chipping but cracks appear at the corners, which indicates that when cracks occur in the base concrete, the fibers are transferred to bear the load in that specific area. This further demonstrates the bridging effect of steel fibers, as shown in Fig. 8f. Moreover, in the macroscopic failure diagram of the fiber-containing groups, the addition of steel fibers creates a lateral constraint on the specimen. This “ring hoop” effect significantly reduces the number of large pieces of the matrix that break off, resulting in only pieces at the edges break off, revealing a “cracked but not broken” state. There is visible peeling and skinning on the surface of the specimen, and the specimen retains more material after destruction than the controls. The hooked-end steel fiber UHPC exhibits a more prominent crack development phenomenon than the straight-steel-fiber UHPC, which also reflects that the number of hooked-end steel fiber is less than that of straight fibers at the same dosage. Hence, the compressive strength of the hooked-end steel fiber UHPC is less than that of the straight-fiber UHPC. Further, the comparison of the failure mode in Fig. 8b, d and in c, e show that the nano-filling effect of PN makes the matrix of UHPC more compact and the resistance of UHPC to failure dramatically improves. This is macroscopically manifested by the spalling of the specimen surface only at the edges and the development of cracks, while the central part of the specimen surface is intact and no cracking phenomenon occurs. This demonstrates that the filling effect of PN and the lapping effect of steel fibers effectively reduce the number of macroscopic and microscopic cracks. This in turn reduces the number of internal pores and external voids of the specimens, improving the mechanical and physical properties of UHPC.

### Interaction between steel fibers and PN in UHPC

**Figure 9:**
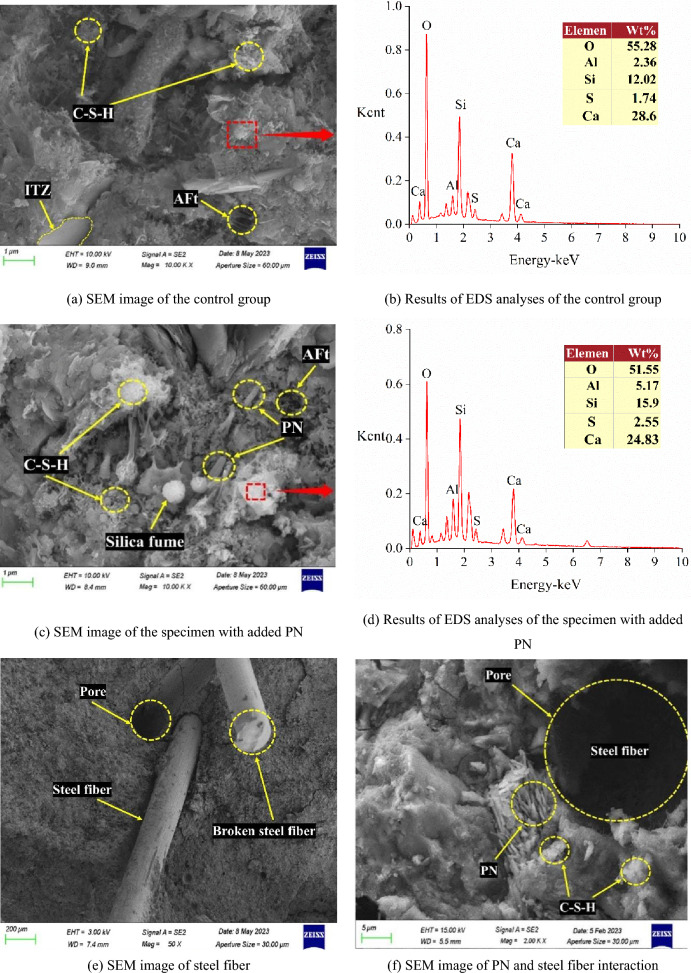
SEM images of the control group and UHPC.

Figure 9 presents the microscopic differences between the control group and the group with PN, the EDS analysis results for the products, the microscopic lap effect of the steel fibers, and the SEM images of the samples with steel fibers and PNs. According to Fig. 9a, c, the SEM image of UHPC with added PN (Fig. 9c) is more intensive than the control group (Fig. 9a), and the generated hydrated calcium silicate gel (depicted as white flocculent in the red box) appears more delicate. This is due to the volcanic ash effect of PN, where active SiO_2_ reacts with the hydration of Ca(OH)_2_ internally, producing more C–S–H gels, as well as the small particle size and large specific surface area of PN, which can fill the internal pores of C–S–H gels. Secondly, a comparison of the hydration products in the yellow circle also reveals that the control in Fig. 9a has less generation and ITZ is visible. Whereas there is still a small amount of incompletely consumed silica without ITZ in Fig. 9c. This is due to the XRD pattern of PN (Fig. 2c) shows it contains a large amount of reactive silica inside, hence blending into UHPC can provide the matrix with raw materials to promote secondary hydration, which is why the hydration products in the microscopic morphology are richer and denser. Meanwhile, PN exerts an effective microscopic enhancement effect. This is one of the reasons why PN addition improves the mechanical properties and increases the matrix density of UHPC. Furthermore, the EDS spectral analysis results in Fig. 9b, d show that the red boxed area of the figure is the hydrated calcium silicate gel. Moreover, the lower Ca/Si ratio in Fig. 9d compared to that in Fig. 9b also illustrates that PN reduces the alkalinity inside the UHPC specimen and the specific weight of Ca(OH)_2_ within the matrix^56^. This is imputed to the low strength and poor bonding of Aft (Ettringite) in an alkaline environment, contributing to a decrease in the overall compactness of UHPC. This further means that the addition of PN reduces the water absorption capacity and porosity of UHPC. In addition, a comparison of Fig. 9e, f shows that the lap action of steel fibers results in the formation of a dense fiber mesh structure in the UHPC matrix, which improves the strength of the UHPC. Moreover, the addition of PN promotes the generation of hydration products that attach to the steel fibers and effectively fill the microscopic cracks near the steel fibers. The double bridging effect of the two materials increases the UHPC compactness, which effectively enhances the mechanical and physical properties of UHPC.

## Conclusions

In this study, the effects of steel fiber shape and dosage, as well as the addition of PN, on the mechanical and physical properties of UHPC were investigated. The following conclusions were drawn.

1. Both straight and hooked-end steel fibers had a remarkable effect on the mechanical properties of UHPC. Straight fibers, with a smaller length-to-diameter ratio, are more effective in improving the compressive strength of UHPC, while hooked-end fibers, with a longer length-to-diameter ratio, are more effective in enhancing the flexural strength of UHPC. The compressive strength of UHPC with a 1% dosage of straight steel fibers was 130.13 MPa, while the flexural strength of UHPC with a 1.5% dosage of straight steel fibers was 25.6 MPa.
2. The two different shapes of steel fibers reduced the water absorption capacity and porosity of UHPC. The effect of straight steel fibers was more significant than that of hooked-end fibers at a 1% steel fiber dosage. The results indicated that the water absorption capacity and porosity of straight-steel fiber UHPC with 1% steel fiber dosage were 33 and 26.7% lower than those of the reference group, respectively. In comparison, when hooked-end steel fiber was used, the water absorption capacity and porosity were 31.5 and 25.9% lower, respectively, than the control specimen.
3. The nano-filling effect of PN and the pro-volcanic ash effect could further refine the internal structure of UHPC. Thus, PN could enhance the macroscopic mechanical and physical properties within a certain dosage range. However, the higher water absorption and more obvious clustering effect of 0.3% PN can lead to defects within the matrix. Therefore, the recommended PN dosage is 0.2%.
4. The brittle “crush” phenomenon exhibited by the control group was mitigated by the combined effect of PN and steel fibers, changing the failure mode of UHPC from brittle to ductile. In addition, specimens with steel fibers and PNs had fewer surface chips falling after crushing.

## The limitation of the study

This study had a potential limitation: due to financial and equipment constraints, we used SEM and EDS-assisted analyses to illustrate the bonding capacity between the PN with the matrix, thus obtained results have some limitations, which is not accurate as the TG nor the TEM methods. We will use both of these approaches for in-depth exploration in subsequent studies.

## Data Availability

All data generated or analyzed during this study are included in this article, the data used in this paper are original data. And the datasets used and/or analyzed during the current study available from the corresponding author on reasonable request.
